# Control of Allergic Rhinitis and Asthma Test (CARAT) can be used to assess individual patients over time

**DOI:** 10.1186/2045-7022-2-16

**Published:** 2012-08-30

**Authors:** Joao A Fonseca, Luis Nogueira-Silva, Mario Morais-Almeida, Ana Sa-Sousa, Luis F Azevedo, Jose Ferreira, Manuel Branco-Ferreira, Rodrigo Rodrigues-Alves, Antonio Bugalho-Almeida, Jean Bousquet

**Affiliations:** 1 Faculdade de Medicina da Universidade do Porto, Health Information and Decision Sciences Department, Porto, Portugal; 2 CINTESIS – Center for Research in Health Technologies and Information Systems, Porto, Portugal; 3 Centro Hospitalar S. João, EPE, Allergy and Clinical Immunology Division, Porto, Portugal; 4 Hospital and Institute CUF, Allergy Unit, Porto, Portugal; 5 Centro Hospitalar S. João, EPE, Internal Medicine Department, Porto, Portugal; 6 Hospital CUF-Descobertas, Allergy and Clinical Immunology Division Unit, Lisboa, Portugal; 7 Faculdade de Medicina da Universidade de Lisboa, Clínica Universitária de Pneumologia, Lisboa, Portugal; 8 Centro Hospitalar de Vila Nova de Gaia/Espinho, EPE, Allergy and Clinical Immunology Division, VN Gaia, Portugal; 9 Centro Hospitalar Lisboa Norte, EPE, Division of Immunoallergology, Lisboa, Portugal; 10 Hospital do Divino Espírito Santo, EPE, Immunoallergology Division, Ponta Delgada, Portugal; 11 Hôpital Arnaud de Villeneuve, Centre Hospitalier Universitaire, Montpellier, France

**Keywords:** Asthma, Allergic rhinitis, Control, Questionnaire

## Abstract

**Background:**

The Control of Allergic Rhinitis and Asthma Test (CARAT10) has been proposed as the first tool to implement the Allergic Rhinitis and its Impact on Asthma initiative guidelines in clinical practice. To serve this purpose, it must have adequate properties to assess the control of an individual over time. This study aimed to prospectively assess the test-retest reliability, responsiveness and longitudinal validity of CARAT10.

**Methods:**

Adults with asthma and allergic rhinitis were enrolled at 4 outpatient clinics of Portuguese central hospitals. At each of the two visits, 4 to 6 weeks apart, patients filled out CARAT10 and additional questionnaires, followed by a medical evaluation blinded to the questionnaires’ answers.

**Results:**

From the 62 patients included, 51 patients completely filled out CARAT10 at both visits. The test-retest reliability, computed as an intra-class correlation coefficient, was 0.82. Regarding responsiveness, a significant change (p = 0.002) of CARAT10 score in clinically unstable patients was observed (95%CI -5.08; -1.31) and the Guyatt’s responsiveness index was 1.54. As for the longitudinal validity assessment, the correlation coefficients of the changes of CARAT10 scores with those of ACQ5 and symptoms VAS ranged from 0.49 to 0.65, while with the physician assessment of control they ranged from 0.31 to 0.41.

**Conclusion:**

CARAT10 has good test-retest reliability, responsiveness and longitudinal validity. It can be used to assess control of allergic rhinitis and asthma, both to compare groups in clinical studies and to evaluate individual patients in clinical practice.

## Introduction

Rhinitis and asthma are highly prevalent diseases that are closely associated. A few questionnaires have been developed and validated to assess the control of rhinitis [1,2] and asthma [3,4]. For over a decade, the Allergic Rhinitis and its Impact on Asthma (ARIA) initiative has recommended the simultaneous assessment and management of these diseases [5,6]. Recent observational studies reinforced the association of rhinitis and asthma. In a cross-sectional study of asthma patients from 85 primary care practices in the United Kingdom, self-reported rhinitis was identified as a major predictor of poor asthma control [7]. Also, data from the West Sweden Asthma Study linked the degree of rhinitis with the risk of having multi-symptom asthma [8].

We have previously developed the Control of Allergic Rhinitis and Asthma Test – a 17-item version [9] – employing a formal methodological approach to ensure its quality and content validity [10]. Subsequently, we conducted a cross-sectional study, in which we performed factor analysis to reduce the questionnaire and assess its unidimensionality, resulting in a simple 10-item version (CARAT10) [11]. In that study, we described how 2 independent factors were extracted from the reduced version, matching the initial theoretical subdomains of asthma and allergic rhinitis. Nevertheless, we demonstrated that CARAT10 has good internal consistency, similar to that of widely used asthma control questionnaires, thus supporting the concept of assessing allergic rhinitis and asthma simultaneously (construct/structural validity). Finally, we also showed that CARAT10 has good discriminative properties and concurrent validity [11].

CARAT10 has been proposed as the first tool to implement ARIA guidelines in clinical practice [12]. To serve this purpose, such tool must have adequate properties to assess asthma and allergic rhinitis control of an individual over time.

Thus, this study aimed to prospectively assess the test-retest reliability, responsiveness and longitudinal validity of CARAT10.

## Methods

### Study design and setting

This prospective observational study comprised two visits, 4 to 6 weeks apart, and was conducted in the first semester of 2009.

Patients were enrolled in 4 allergy outpatient clinics of central hospitals in three Portuguese regions – north (Porto and Gaia), south (Lisbon) and the Azores islands (Ponta Delgada).

The study was approved by the Hospital S. João review board (Comissão de Ética para a Saúde) and was given the project nº 120/08. Each patient gave his/her written informed consent.

### Participants

All patients between 18 and 70 years of age, with a medical diagnosis of asthma and allergic rhinitis and at least 6 months of follow-up at the clinic were eligible. Only patients unable to fill the questionnaire were excluded.

### Data collection

At each visit, patients were asked to fill CARAT10 and additional tools: the Asthma Control Questionnaire (ACQ5) [3] and three visual analogue scales (VAS) concerning all airways symptoms, bronchial/pulmonary symptoms and nasal symptoms as measures of self-perceived control. They also underwent lung function tests followed by a medical evaluation. The study design is described in the Additional file 1: Figure S1.

Lung function and exhaled nitric oxide (FeNO_50_) were measured according to the 2005 ATS/ERS position statements [13,14]. Lung function variables included the forced vital capacity (FVC), the forced expiratory volume in 1 second (FEV1) and the peak expiratory flow (PEF). FeNO_50_ was evaluated only in two of the four centres and expressed as parts per billion (ppb).

The medical evaluation was carried out by the attending allergy specialist, who was blinded to the questionnaires’ answers. The physician classified the patient’s rhinitis and asthma severity and rated the rhinitis and asthma control in two 10 cm VAS, according to his/her judgment and bearing in mind the classifications of ARIA [5] and Global Initiative for Asthma (GINA) [15]. The treatment decisions were made by the attending physician, based on the clinical history, physical examination and lung function following ARIA and GINA guidelines. In addition, known allergies and current medication, as well as the decision regarding treatment plan (increase, maintain or decrease treatment) were registered. In the second visit, the same procedures were repeated and, additionally, the physician filled out two 10 cm VAS regarding the variation in asthma control and in rhinitis control (from 0 - greatest worsening, to 10 - greatest improvement).

### Statistical analysis

The statistical analysis was carried out using SPSS 19.0 (SPSS Inc., Chicago, IL, USA). The level of significance was set at p < 0.05. The population was described using standard descriptive statistical techniques.

We derived dichotomous variables, classifying the patient as having controlled or not controlled asthma and controlled or not controlled rhinitis, from the physician’s control VAS. Patients were categorized as controlled if the physician filled out the VAS in the ]6,10]cm interval.

Patients who were graded between 5.0 and 6.0 in the second visit’s control variation VAS were considered to be clinically stable [3].

We classified the patients’ rhinitis severity according to the ARIA recommendations and the asthma control according to the GINA guidelines. The results from both visits were pooled and were plotted against the scores in CARAT10 and its factors.

#### Properties of CARAT10

The CARAT10 internal consistency analysis comprised the assessment of its internal consistency with Cronbach’s α.

The test-retest reliability was assessed using intra-class correlation coefficient (ICC) in clinically stable patients between visits [16].

The concurrent validity was studied using Spearman’s correlation coefficients between CARAT10/CARAT factors and 1) control assessment instruments or 2) physician’s assessment.

The responsiveness of CARAT10 was evaluated in the group of clinically unstable patients – those with either unstable asthma, unstable rhinitis, or both. We used a paired T-test to assess the within-patient change in the CARAT10 score. Moreover, the Guyatt’s responsiveness index (GRI) was calculated as [17,18]:

(1) $$GRI\frac{\text{mean change of CARAT 10 in the unstable group}}{\text{SD of change CARAT 10 in the stable group}}$$

The longitudinal validity was assessed by Spearman’s correlation coefficients calculated between the variation of the CARAT10 score and the variation of the other measures.

For hypotheses testing, *a priori* predictions for the correlation coefficients were, based on previous studies as follows: (i) 0.6–0.8 with ACQ5; (ii) 0.6–0.8 with the symptoms VAS; (iii) 0.4–0.6 with the physician’s assessment [3,10].

## Results

We included 62 patients; one patient did not attend the 2^nd^ visit. Seven patients in the first visit and 3 in the second incompletely filled out CARAT10 (frequencies of unanswered questions in the Additional file 1: Table S1); 51 patients completely filled out the CARAT10 questionnaire in both visits. Table 1 summarizes the sample’s characteristics, physician assessment, lung function test results and scorings for the several questionnaires. Table 2 shows the CARAT10 scores according to the patients’ self-perceived control. Scores and subscores of CARAT10 in both visits, in patients with controlled and uncontrolled rhinitis and asthma, are presented in the Additional file 1: Table S2.

**Table 1:** – **Sample’s characteristics and results of physician assessment, control questionnaires and lung function tests**

	**1**^**st **^**visit *n = 62***	**2**^**nd **^**visit *n = 61***
**Gender** Female n (%)	37 (60)	36 (59)
**Age** mean (SD) years	39.6 (14.5)	39.8 (14.5)
**Physician assessment** n (%)		
* Asthma severity*		
Intermittent	12 (19)	7 (12)
Mild persistent	21 (34)	16 (28)
Moderate persistent	24 (39)	27 (48)
Severe persistent	5 (8)	7 (12)
* Rhinitis classification*		
Intermittent	15 (28)	16 (34)
Persistent	38 (72)	31 (66)
Mild	27 (51)	26 (58)
Moderate/severe	26 (49)	19 (42)
* Control*		
Both controlled	24 (39)	33 (59)
Only asthma controlled	15 (24)	8 (14)
Only rhinitis controlled	8 (13)	6 (11)
Both uncontrolled	15 (24)	9 (16)
* Treatment decision*		
Reduce	3 (5)	2 (4)
Maintain	29 (47)	42 (75)
Increase	30 (48)	12 (21)
**ACQ5 score** median (P25-P75)	1 (0.2-2.5)	0.8 (0-2.2)
<0.5 n (%)	22 (35)	26 (42)
0.5–1.5 n (%)	14 (23)	12 (20)
>1.5 n (%)	26 (42)	23 (38)
VAS* median (P25-P75)		
All symptoms	5 (2-7)	4 (2-6.5)
Bronchial/pulmonary symptoms	4 (2-7)	3 (2-6)
Nasal symptoms	5.7 (3-8)	3 (2-7)
**Lung function** mean (sd)^**#**^		
FVC	102 (17.1)	100 (20.8)
FEV_1_	92 (19.7)	90 (23)
PEF	89 (25)	88 (26.7)
**FeNO**_**50**_^**ª**^	30 (23.1)	24 (21.7)

ACQ5 – Asthma control questionnaire (5-question); VAS – visual analogue scale; FVC – forced vital capacity; FEV_1_ – forced expiratory volume in 1 second; PEF – peak expiratory flow; FeNO_50_- fractional exhaled nitric oxide.

*1-10, 1 being no disturbance; # 1^st^ visit n = 55; 2^nd^ visit n = 53; ª 1^st^ visit n = 23; 2^nd^ visit n = 31.

**Table 2:** – **Comparison of CARAT10 scores according to the patients’ self-perceived control**

		**Controlled‡**		**Not controlled**		**Total**	
		**mean (SD)**	**[min-max]**	**mean (SD)**	**[min-max]**	**mean (SD)**	**[min-max]**
**1**^**st **^**visit**	CARAT10 *	22 (4.93)	[11-29]	12.3 (5.91)	[3-24]	18 (7)	[3-29]
	Rhinitis subscore #	7.5 (2.89)	[2-11]	3.1 (2.57)	[0-10]	5.2 (3.49)	[0-11]
	Asthma subscore ª	14.9 (2.41)	[9-18]	8.3 (4.87)	[1-18]	12.7 (4.67)	[1-18]
**2**^**nd **^**visit**	CARAT10*	23.4 (5.12)	[10-30]	14.8 (7.61)	[4-27]	20.4 (7.32)	[4-30]
	Rhinitis subscore #	8.3 (2.64)	[2-12]	4.5 (3.24)	[1-11]	7.2 (3.33)	[1-12]
	Asthma subscore ª	15 (3.13)	[6-18]	8.8 (4.53)	[3-17]	13.4 (4.48)	[3-18]

‡ The classification of self-preceived control was established with the patients’ all symptoms VAS for CARAT10, with the nasal symptoms VAS for the rhinitis subscore and with the bronchial/pulmonary symptoms VAS for the asthma subscore. * Range 0-30; # Range 0-12; ª Range 0-18.

Seventeen patients (28%) were classified as having clinically stable asthma and rhinitis. The unstable group included 30 patients with both unstable asthma and rhinitis, 9 only with unstable asthma and 5 only with unstable rhinitis.

**Figure 1:**
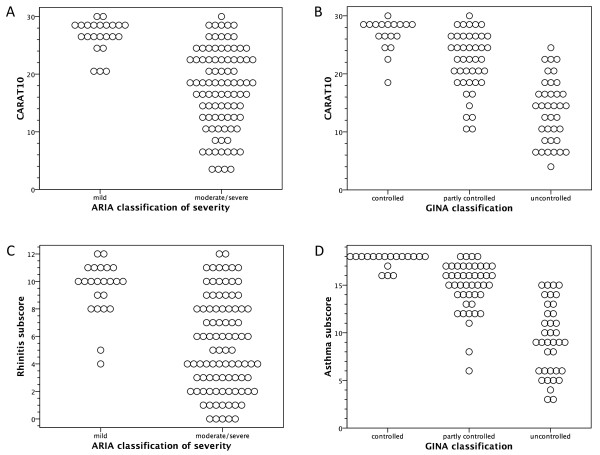
CARAT10 and its subscores plotted against the classifications of rhinitis severity and asthma control, according to ARIA recommendations and GINA guidelines.

The plots of CARAT10 scores according to the guidelines’ classifications of rhinitis severity and asthma control are shown in Figure 1.

### Properties of CARAT10

Regarding internal consistency, the Cronbach’s α was 0.84 for CARAT10, 0.76 for the rhinitis subscore and 0.80 for the asthma subscore. As for the test-retest reliability, an ICC of 0.82 was found in the stable group. Regarding concurrent validity, all the correlation coefficients between CARAT10/CARAT factors and 1) control assessment instruments or 2) physician’s assessment met the *a priori* predictions and were statistically significant with p < 0.001 (Table 3).

**Table 3:** – **Spearman’s correlations of CARAT10 and its factors with external measures of rhinitis and asthma control in the first visit **

		**Symptoms VAS**			**Physician assessment**	
	**ACQ5**	**All symptoms**	**Bronchial/pulmonary symptoms**	**Nasal symptoms**	**Asthma control**	**Rhinitis control**
CARAT10	−0.79	**−0.76**	−0.75	−0.69	0.58	0.34*
Rhinitis subscore	−0.50	−0.59	−0.51	**−0.70**	0.35*	**0.43**
Asthma subscore	**−0.78**	−0.62	**−0.71**	−0.49	**0.65**	0.24#

The correlations between the measures of similar domains are shown in bold.

All correlations coefficients met the *a priori* predictions and were statistically significant with p < 0.001, except * p < 0.01 and # p = 0.073.

Concerning responsiveness, we observed a significant within-patient change of CARAT10 scoring in clinically unstable patients (95% confidence interval [-5.08; -1.31], p = 0.002). The Guyatt’s responsiveness index was 1.54.

The correlation coefficients of the measurements variations between visits ranged from 0.49 to 0.65 for ACQ5 and the symptoms VAS, while for the physician assessment of control the coefficients ranged from 0.31 to 0.41. Several correlation coefficients of external measures and CARAT subscores were lower than the *a priori* predictions, but only one was below 0.4 [10]. These results are summarized in Table 4.

**Table 4:** – **Longitudinal validity – comparison of the variation of CARAT10 with the variation of external measures of control **

		**Symptoms VAS**			**Physician assessment**	
	**ΔACQ5**	**ΔAll symptoms**	**ΔBronchial/pulmonary symptoms**	**ΔNasal symptoms**	**ΔAsthma control**	**ΔRhinitis control**
ΔCARAT10	−0.63	**−0.65**	−0.52	−0.53	0.45*	0.31#
ΔRhinitis subscore	−0.51	−0.52	−0.53	**−0.56**‡	0.44*	0.41*
ΔAsthma subscore	**−0.55**‡	−0.60	**−0.49**‡	−0.28#	**0.31#**‡	0.24ª

The correlations between the measures of similar domains are shown in bold.

All correlations were statistically significant with p < 0.001, except * p < 0.01, # p < 0.05 and ª p = 0.09. ‡ did not meet the *a priori* predictions for the correlation coefficients: 1) 0.6–0.8 with ACQ5 and with the symptoms VAS; 2) 0.4–0.6 with the physician’s assessment.

**Figure 2:**
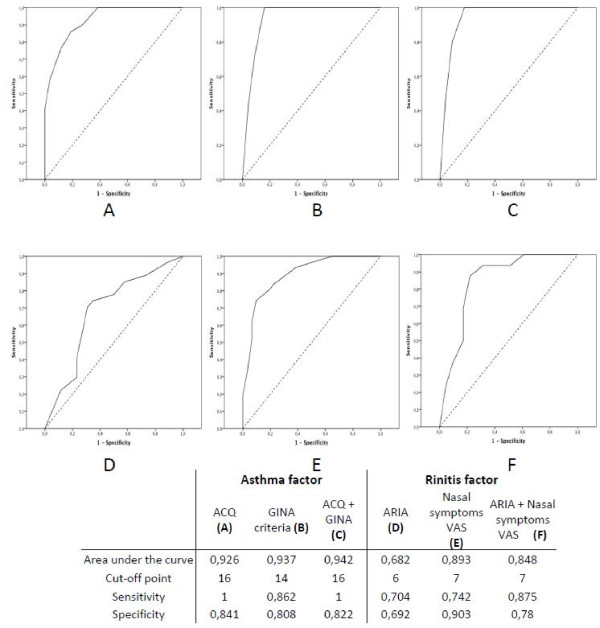
Receiver operating characteristic curves and diagnostic test properties of CARAT10’ with composite score of (A) ACQ score and Nasal VAS, (B), GINA and ARIA criteria and (C) ACQ, GINA, Nasal VAS and ARIA.

**Figure 3:**
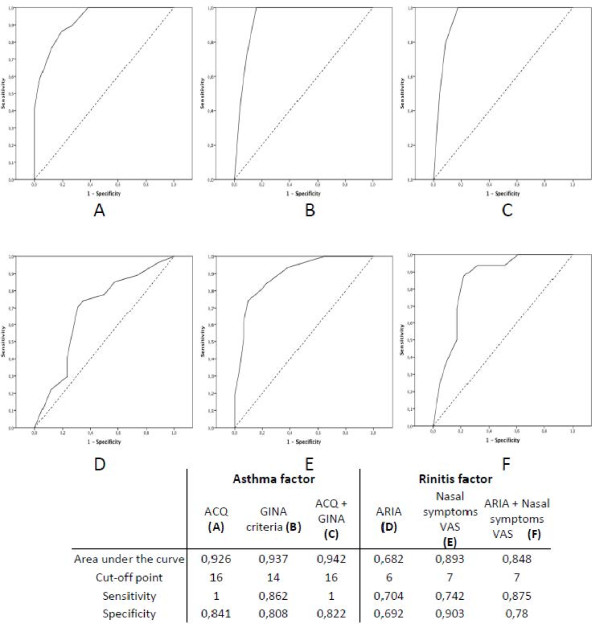
Receiver operating characteristic curves and diagnostic test properties of CARAT10’ asthma factor with (A) ACQ score, (B) GINE criteria, and (C) a composite score of GINA and ACQ; and CARAT10’ rhinitis factor with (D) ARIA classification, (E) the nasal symptoms VAS and (F) a composite score of ARIA and the symptoms VAS.

Receiver operating characteristic (ROC) curves were plotted, comparing CARAT10’ scores (Figure 2) and subscores (Figure 3) with asthma and rhinitis assessment tools. The AUC for CARAT were between 0.941 and 0.948 for CARAT (Figure 2). Higher AUC were observed for the asthma factor (0.926-0.942) than for the rhinitis factor (0.682-0.893) (Figure 3).

## Discussion

The longitudinal assessment of the Control of Allergic Rhinitis and Asthma Test (CARAT10) showed that it has adequate properties to assess individual patients over time.

This is the third phase of a project that aimed to develop and validate a tool to simultaneously assess the control of rhinitis and asthma. We have previously shown that CARAT10 has good internal consistency and internal validity, supporting the concept of a single questionnaire assessing allergic rhinitis and asthma simultaneously, and showing it may be used to compare groups [11]. We had observed good correlations between CARAT10 and ACQ5, symptoms VAS and the physician’s assessment of control, similar to that of other widely used control questionnaires for asthma. The new data confirmed our previous results. As for test-retest reliability, the ICC for CARAT10 scores between visits in stable patients was 0.82, better than the ICC previously reported for Asthma Control Test (ACT) (0.77) [19]. Also, the Guyatt’s responsiveness index observed is somewhat higher than that of ACQ7 (1.35) [3]. Regarding longitudinal validity, like ACT, CARAT10 correlates better with changes in ACQ than with the physician’s assessment; the correlation coefficient of CARAT10 with symptoms VAS was similar to the previously reported correlation of ACQ7 with asthma symptoms [3,19]. The correlation coefficient for changes between visits of CARAT10 with the all symptoms VAS met the *a priori* predictions (Table 4). The correlation coefficients of CARAT10 subscores with external measures of rhinitis and asthma status were lower than predicted. These subscores were defined using standard procedures of exploratory factor analysis in a previous study [11]. However, questions on waking up at night or increasing medication may be related to asthma and/or rhinitis in different patients. Therefore, a reassessment of CARAT10 structure in different populations seems to be necessary.

In this multicenter longitudinal study, it was necessary to categorize patients as clinically stable or unstable between visits. The criterion was the physician’s rating of control variation between visits, since there is no gold standard for the measurement of control of allergic rhinitis and asthma. The intrinsic insufficiencies of physician rating [20] may undermine the results observed. However, the direction of this bias worsens the questionnaire’s properties. Therefore, the estimates for the evaluative and discriminative properties of CARAT10 are conservatively reported.

CARAT10 has been thoroughly studied in cross-sectional and prospective studies and now meets almost all requirements of COSMIN - COnsensus-based Standards for the selection of health Measurement Instruments [21]. Further studies with larger datasets are needed to firmly establish the cut values for the CARAT10 global score and the rhinitis and asthma subscores [22]. The existing data seems to suggest that a CARAT10 score over 24 identifies controlled patients, with a rhinitis subscore over 8 and an asthma subscore equal or greater than 16.

The availability of CARAT10 for clinical practice in multiple countries requires cross-cultural validation in other languages. A website to support an adequate process of translation was developed ( http://www.caratnetwork.org) and researchers are invited to participate. Cross-cultural adaptation of CARAT has started in over 10 different countries [23]. It was organized in 3 phases, following the Global Allergy and Asthma European Network (GA^2^LEN) network recommendations [24]: forward translation, backward translation and patient testing; clinical validation studies for these languages should follow.

In summary, this study showed that CARAT10 has adequate test-retest reliability, responsiveness and longitudinal validity while confirming its high internal consistency and concurrent validity. Therefore, CARAT10 can be used both in clinical studies and in clinical practice, to compare groups and to evaluate individual patients over time.

## Abbreviations

ACQ: Asthma control questionnaire; ACT: Asthma Control Test; ARIA: Allergic Rhinitis and its Impact on Asthma; CARAT10: Control of Allergic Rhinitis and Asthma Test; COSMIN: COnsensus-based Standards for the selection of health Measurement Instruments; FeNO_50_: Fraction of exhaled nitric oxide; FEV1: Forced expiratory volume in 1 second; FVC: Forced vital capacity; GA^2^LEN: Global Allergy and Asthma European Network; GINA: Global Initiative for Asthma; GRI: Guyatt’s responsiveness index; ICC: Intra-class correlation coefficient; PEF: Peak expiratory flow; ROC: Receiver operating characteristic; VAS: Visual analogue scales.

## Competing interests

JAF declares he has been given honoraria for advisory boards and lectures during meetings from GSK, MSD and Novartis. MMA declares he has been given honoraria for advisory boards and lectures during meetings from GSK, MSD AZ, Faes-pharma and Novartis. JB declares he has been given honoraria for scientific and advisory boards, lectures during meetings and press conferences from StallergÃ¨nes, Actelion, Almirall, AstraZeneca, Chiesi, GSK, Merck, MSD, Novartis, OM Pharma, Sanofi-Aventis, Schering Plough, Teva, Uriach. The remaining authors declare that they have no competing interests.

## Authors' contributions

JAF is responsible for the CARAT project, guarantor of the paper and participated in all stages and tasks, LNS participated in the analysis and writing the manuscript draft, ASS participated in data collection and analysis and collaborated in the process of writing the manuscript draft, MMA and ABA participated in study design and reviewed the manuscript; LFA participated in study design, led the data analysis and reviewed the manuscript; JF, MBF, RRA participated in the data collection and reviewed the manuscript and JB provided critical review during the project and reviewed the manuscript. All authors read and approved the final manuscript.

## Supplementary Material

Additional file 1 **Table S1.** Frequencies of unanswered questions of CARAT 10 in each visit. **Table S2** - Scores and subscores of CARAT 10 in both visits in patients with controlled and uncontrolled rhinitis and asthma. **Figures S1** - Study design - data collection and properties evaluation. Click here for file

## References

[B1] Schatz M Meltzer EO Nathan R Derebery MJ Mintz M Stanford RH Psychometric validation of the rhinitis control assessment test: a brief patient-completed instrument for evaluating rhinitis symptom control Ann Allergy Asthma Immunol 2010 104 2 118 24 10.1016/j.anai.2009.11.063 20306814

[B2] Demoly P Jankowski R Chassany O Bessah Y Allaert FA Validation of a self-questionnaire for assessing the control of allergic rhinitis Clin Exp Allergy 2011 41 6 860 8 10.1111/j.1365-2222.2011.03734.x 21518040

[B3] Juniper EF O'Byrne PM Guyatt GH Ferrie PJ King DR Development and validation of a questionnaire to measure asthma control Eur Respir J 1999 14 4 902 7 10.1034/j.1399-3003.1999.14d29.x 10573240

[B4] Nathan RA Sorkness CA Kosinski M Schatz M Li JT Marcus P Development of the Asthma Control Test: a survey for assessing asthma control J Allergy Clin Immunol 2004 113 1 59 65 10.1016/j.jaci.2003.09.008 14713908

[B5] Bousquet J Khaltaev N Cruz AA Denburg J Fokkens WJ Togias A Allergic Rhinitis and its Impact on Asthma (ARIA) 2008 update (in collaboration with the World Health Organization, GA2LEN and AllerGen) Allergy: Eur J Allergy Clin Immunol 2008 63 SUPPL. 86 8 160 10.1111/j.1398-9995.2007.01620.x 18331513

[B6] Brozek JL Bousquet J Baena-Cagnani CE Bonini S Canonica GW Casale TB Allergic Rhinitis and its Impact on Asthma (ARIA) guidelines: 2010 revision J Allergy Clin Immunol 2010 126 3 466 76 10.1016/j.jaci.2010.06.047 20816182

[B7] Clatworthy J Price D Ryan D Haughney J Horne R The value of self-report assessment of adherence, rhinitis and smoking in relation to asthma control Prim Care Respir J 2009 18 4 300 5 10.4104/pcrj.2009.00037 19562233 PMC6619365

[B8] Lotvall J Ekerljung L Lundback B Multi-symptom asthma is closely related to nasal blockage, rhinorrhea and symptoms of chronic rhinosinusitis - evidence from the West Sweden Asthma Study Respir Res 2010 11 1 163 10.1186/1465-9921-11-163 21110834 PMC3004848

[B9] Nogueira-Silva L Martins SV Cruz-Correia R Azevedo LF Morais-Almeida M Bugalho-Almeida A Control of allergic rhinitis and asthma test–a formal approach to the development of a measuring tool Respir Res 2009 10 52 52 19534774 10.1186/1465-9921-10-52 PMC2706215

[B10] Van Oene CM Van Reij EJF Sprangers MAG Fokkens WJ Quality-assessment of disease-specific quality of life questionnaires for rhinitis and rhinosinusitis: a systematic review Allergy: European Journal of Allergy and Clinical Immunology 2007 62 12 1359 71 10.1111/j.1398-9995.2007.01482.x 17983371

[B11] Fonseca JA Nogueira-Silva L Morais-Almeida M Azevedo L Sa-Sousa A Branco-Ferreira M Validation of a questionnaire (CARAT10) to assess rhinitis and asthma in patients with asthma Allergy 2010 65 8 1042 8 10.1111/j.1398-9995.2009.02310.x 20121755

[B12] Bousquet J Schunemann HJ Zuberbier T Bachert C Baena-Cagnani CE Bousquet PJ Development and implementation of guidelines in allergic rhinitis - an ARIA-GA2LEN paper Allergy 2010 65 10 1212 21 10.1111/j.1398-9995.2010.02439.x 20887423

[B13] Miller MR Hankinson J Brusasco V Burgos F Casaburi R Coates A Standardisation of spirometry Eur Respir J 2005 26 2 319 38 10.1183/09031936.05.00034805 16055882

[B14] ATS/ERS recommendations for standardized procedures for the online and offline measurement of exhaled lower respiratory nitric oxide and nasal nitric oxide Am J Respir Crit Care Med 2005 171 8 912 930 15817806 10.1164/rccm.200406-710ST

[B15] Bateman ED Hurd SS Barnes PJ Bousquet J Drazen JM FitzGerald M Global strategy for asthma management and prevention: GINA executive summary Eur Respir J 2008 31 1 143 78 10.1183/09031936.00138707 18166595

[B16] Streiner DL Norman GR Health measurement scales: a practical guide to their development and use 1995 2 Oxford; New York: Oxford University Press

[B17] Guyatt G Walter S Norman G Measuring change over time: assessing the usefulness of evaluative instruments J Chronic Dis 1987 40 2 171 8 10.1016/0021-9681(87)90069-5 3818871

[B18] Schmitt JS Di Fabio RP Reliable change and minimum important difference (MID) proportions facilitated group responsiveness comparisons using individual threshold criteria J Clin Epidemiol 2004 57 10 1008 18 10.1016/j.jclinepi.2004.02.007 15528051

[B19] Schatz M Sorkness CA Li JT Marcus P Murray JJ Nathan RA Asthma Control Test: reliability, validity, and responsiveness in patients not previously followed by asthma specialists J Allergy Clin Immunol 2006 117 3 549 56 10.1016/j.jaci.2006.01.011 16522452

[B20] Juniper EF Chauhan A Neville E Chatterjee A Svensson K Mörk AC Clinicians tend to overestimate improvements in asthma control: An unexpected observation Prim Care Respir J 2004 13 4 181 4 10.1016/j.pcrj.2004.04.003 16701667 PMC6750691

[B21] Mokkink LB Terwee CB Patrick DL Alonso J Stratford PW Knol DL The COSMIN checklist for assessing the methodological quality of studies on measurement properties of health status measurement instruments: an international Delphi study Qual Life Res 2010 19 4 539 49 10.1007/s11136-010-9606-8 20169472 PMC2852520

[B22] Juniper EF Bousquet J Abetz L Bateman ED Identifying 'well-controlled' and 'not well-controlled' asthma using the Asthma Control Questionnaire Respir Med 2006 100 4 616 21 10.1016/j.rmed.2005.08.012 16226443

[B23] Fonseca JA, Correia De Sousa J, Sá-Sousa A, Burnay E, Tsiligianni I, Nogueira-Silva L Cross-cultural adaptation of Control of Rhinitis and Asthma Test (CARAT) 2011 Amsterdam, The Netherlands: 2nd IPCRG Scientific Meeting

[B24] Braido F Bousquet PJ Brzoza Z Canonica GW Compalati E Fiocchi A Specific recommendations for PROs and HRQoL assessment in allergic rhinitis and/or asthma: a GA(2)LEN taskforce position paper Allergy 2010 65 8 959 968 Annex 3 10.1111/j.1398-9995.2010.02383.x 20486919

