# Supplementary material for: Control of Allergic Rhinitis and Asthma Test (CARAT) can be used to assess individual patients over time
Source: Clin Transl Allergy. 2012 Aug 30;2:16. doi: 10.1186/2045-7022-2-16 (PMC3520832; doi:10.1186/2045-7022-2-16)
Supplement: Additional file 1 — Table S1. Frequencies of unanswered questions of CARAT 10 in each visit. Table S2 - Scores and subscores of CARAT 10 in both visits in patients with controlled and uncontrolled rhinitis and asthma. Figures S1 - Study design - data collection and properties evaluation. [file 2045-7022-2-16-S1.pdf]

Control of Allergic Rhinitis and Asthma Test (CARAT) can be used to assess individual patients over time

Additional file

Table 1 - Frequencies of unanswered questions of CARAT10 in each visit.

	**Number of Missing answers**	
	1st visit	2nd visit
Q1 - Blocked nose?	0	1
Q2 - Sneezing?	0	2
Q3 - Itchy nose?	0	2
Q4 - Runny nose?	0	1
Q5 - Shortness of breath/dyspnea?	1	1
Q6 - Wheezing in the chest?	0	1
Q7 - Chest tightness upon physical exercise?	0	2
Q8 - Tiredness/ limitations in doing daily tasks?	6	1
Q9 - Woke up during the night?	0	1
Q10 - Increase in the use of medicines…?	0	1

Table 2 - Scores and subscores of CARAT10 in both visits in patients with controlled and uncontrolled rhinitis and asthma.

		1st visit			2nd visit		
		CARAT10 (max 30)	Rhinitis subscore (max 12)	Asthma subscore (max 18)	CARAT10 (max 30)	Rhinitis subscore (max 12)	Asthma subscore (max 18)
**all**	Mean	17,9	5,2	12,7	20,4	7,2	13,4
	SD	7.18	3,5	4,67	7.32	3,3	4,5
	Min (n)	3 (1)	0 (5)	1 (1)	4 (1)	1 (3)	3 (1)
	Max (n)	29 (2)	11 (5)	18 (6)	30 (3)	12 (4)	18 (13)
	n	55	62	55	58	59	60
**both controlled**	Mean	23,35	6,96	15,95	23,29	8,16	15,15
	SD	4,03	3,13	1,73	5,95	2,72	3,65
	Min (n)	15 (1)	0 (1)	12 (1)	6 (1)	2 (1)	4 (1)
	Max (n)	29 (2)	11 (3)	18 (6)	30 (3)	12 (3)	18 (12)
	n	20	24	20	31	31	33
**uncontrolled rhinitis**	Mean	18,15	4,4	13,46	18,75	7,75	11
	SD	4,95	3,52	2,9	6,73	3,28	4,5
	Min (n)	11 (1)	0 (2)	9 (2)	11 (1)	4 (2)	5 (1)
	Max (n)	28 (1)	11 (2)	17 (3)	28 (2)	12 (1)	17 (1)
	n	13	15	13	8	8	8
**uncontrolled asthma**	Mean	11,43	5,38	6,71	16	6,5	10,2
	SD	6,83	3,2	4,96	7,38	3,56	4,21
	Min (n)	3 (1)	2 (1)	1 (1)	7 (1)	2 (2)	5 (1)
	Max (n)	23 (1)	10 (2)	14 (1)	24 (1)	10 (1)	15 (1)
	n	7	8	7	5	6	5
**both uncontrolled**	Mean	13,6	3,27	10,33	14,1	3,6	10,6
	SD	7,2	3,08	4,76	6,9	2,8	4,5
	Min (n)	4 (2)	0 (2)	2 (1)	4 (1)	1 (2)	3 (1)
	Max (n)	27 (1)	10 (1)	17 (1)	27 (1)	10 (1)	17 (1)
	n	15	15	15	9	9	9

SD – standard deviation; max – maximum score; min – minimum score

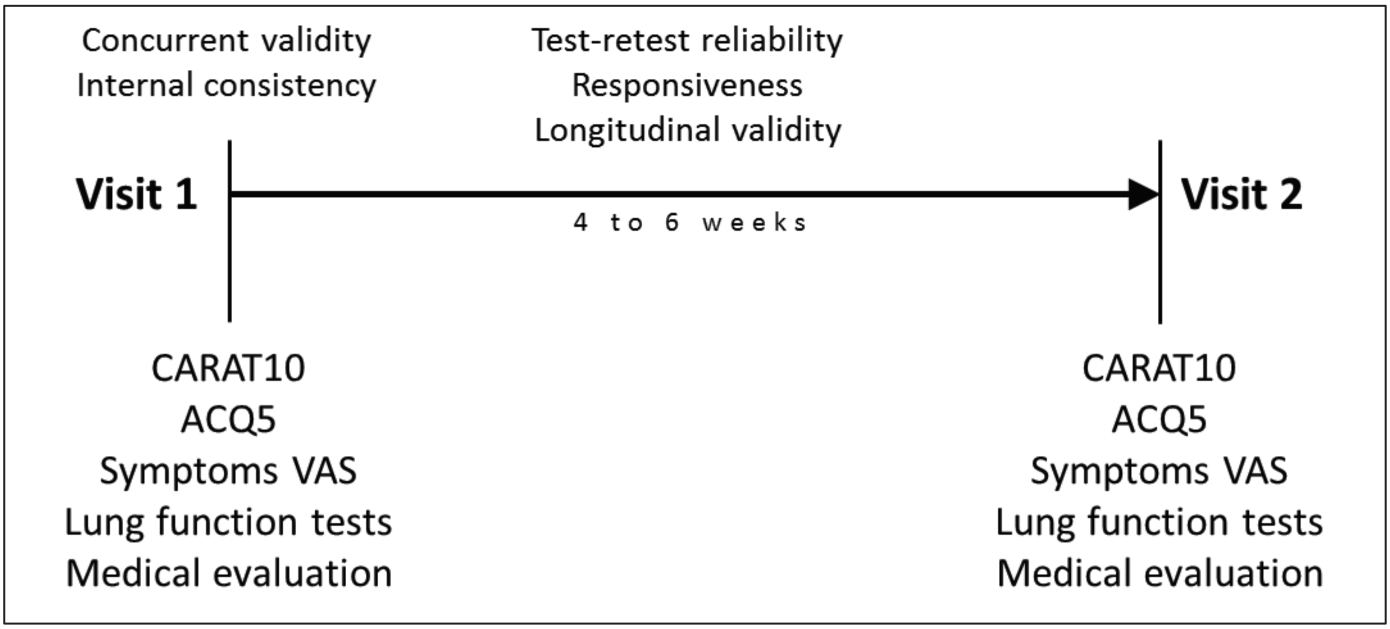

Figure 1 – Study design – data collection and properties evaluation.
